# GD2-targeted immunotherapy in pediatric bone sarcomas: a systematic review of emerging strategies and combination approaches

**DOI:** 10.3389/fimmu.2026.1895000

**Published:** 2026-08-04

**Authors:** Elisa Tirtei, Sofia Poggi Longostrevi, Sabrina Bombaci, Federico Mercolini, Maria Grazia Pionelli, Sebastian Dorin Asaftei, Franca Fagioli

**Affiliations:** 1 Pediatric Onco-Hematology Department, Regina Margherita Children’s Hospital, Turin, Italy; 2 Department of Public Health and Pediatrics, University of Turin, Turin, Italy; 3 Pediatric Hematology and Oncology, IRCCS Azienda Ospedaliero-Universitaria di Bologna, Bologna, Italy; 4 Pediatric Oncology Unit, Department of Pediatric Onco-Hematology, Santobono-Pausilipon Children’s Hospital, AORN, Naples, Italy

**Keywords:** anti-GD2, GD2, osteosarcoma, pediatric bone sarcoma, target therapy

## Abstract

**Introduction:**

Primary bone sarcomas, including Osteosarcoma (OS) and Ewing sarcoma (ES), are rare pediatric malignancies with limited therapeutic advances over recent decades and poor outcomes, especially for patients with metastatic and refractory/relapsed disease. Disialoganglioside 2 (GD2) has emerged as a promising immunotherapeutic target due to its high expression in pediatric bone sarcomas and its role in tumor progression and treatment resistance. This systematic review summarizes current evidence on GD2-targeted therapies and combination strategies in pediatric bone sarcomas.

**Methods:**

A systematic review was conducted according to PRISMA 2020 guidelines using PubMed, Embase, Web of Science, and ClinicalTrials.gov. Preclinical and clinical studies investigating GD2-targeted therapies in pediatric bone sarcomas were included, together with registered clinical trials.

**Results:**

Twenty-six studies met inclusion criteria, including 17 preclinical and 9 clinical studies, alongside 15 ongoing clinical trials. Preclinical evidence consistently demonstrated antitumor activity of GD2-directed approaches, including monoclonal antibodies (mAbs), CAR-T cells, bispecific antibodies, and radio-immunotherapy. Combination strategies, particularly anti-GD2 mAbs with chemotherapy, enhanced antitumor efficacy through induction of apoptosis, activation of endoplasmic reticulum stress pathways, and inhibition of tumor invasiveness. Clinical evidence, although limited and heterogeneous, suggested encouraging activity of Dinutuximab beta-based chemo-immunotherapy, especially in ES. Several clinical trials are ongoing, mainly early-phase and focusing on relapsed or refractory diseases.

**Discussion:**

GD2 represents a biologically relevant and clinically promising target in pediatric bone sarcomas. However, major translational challenges remain, including limited clinical data, heterogeneous study designs, and the lack of standardized assays for GD2 expression assessment. Further collaborative international studies are needed to optimize patient selection and accelerate the development of GD2-targeted combination immunotherapy strategies.

## Introduction

Primary bone sarcomas are tumors of mesenchymal origin which, despite being rare, have a higher incidence among children, adolescents, and young adults (1).

Despite progress in management of localized disease, mainly through a multimodal treatment, including chemotherapy, surgery and/or radiotherapy, clinical outcomes of these tumors remain poor, largely due to high rates of recurrence and to the frequent presence of metastases at diagnosis (2). Survival has changed little over recent decades, reflecting persistent challenges due to limited understanding of tumor biology, development of treatment resistance, and the considerable toxicity associated with current therapies (3).

In this context, increasing interest has been directed towards the identification of tumor-associated antigens as novel biomarkers and therapeutic targets and, among others, Disialoganglioside 2 (GD2) emerged as particularly promising (4).

GD2 is a plasma-membrane sphingolipid that, unlike other gangliosides, exhibits limited expression in normal tissues while being highly expressed across a variety of malignancies (5). Functionally, it has been implicated in cell adhesion, modulation of tumor microenvironment, and immune checkpoint regulation (4).

Among pediatric cancers, GD2 expression is most prominent in neuroblastoma (NB), but elevated levels have also been reported in primary central nervous system tumors and several sarcoma subtypes (4).

Both Osteosarcoma (OS) and Ewing sarcoma (ES), the two most common pediatric bone sarcomas, demonstrate high GD2 expression (4, 5).

Notably, in OS, GD2 expression appears to be further increased in relapsed tumors compared with samples obtained at first diagnosis, suggesting a potential association with chemotherapy resistance and reinforcing its importance as a therapeutic target (6).

Having assessed the relevance of GD2 as an actionable target and the need of new strategies to improve survival of patients with bone sarcomas, we conducted a systematic review with the aim to compile all available evidence on current anti-GD2 strategies and to explore new possible perspectives for the management of pediatric bone sarcomas.

## Materials and methods

A comprehensive literature search was conducted to identify scientific studies investigating the role of GD2 in bone sarcomas, in accordance with the Preferred Reporting Items for Systematic Review and Meta‐Analysis criteria (PRISMA 2020) (7). PubMed (https://pubmed.ncbi.nlm.nih.gov/), Embase (https://www.embase.com), and Web of Science (https://www.webofscience.com) were systematically searched for relevant studies through the following key words: “*bone sarcomas*” OR “*Osteosarcoma*” OR “*Ewing sarcoma*” OR “*pediatric sarcomas*” AND “*GD2*” OR “*Disialoganglioside GD2*” OR “*anti-GD2*”. Complete search strings for each database are reported in **Supplementary Table 1**.

The search strategy combined controlled vocabularies with free-text keywords. No filters on language, publication date, or study design were initially applied, and all records indexed from database inception to search date were considered.

Study selection and quality assessment were conducted independently and in duplicate by four reviewers (E.T., S.P.L., F.M., MG.P.). Inclusion and exclusion criteria applied for appropriate published study selection are summarized in **Table 1**.

**Table 1:** Inclusion and exclusion criteria for literature systematic review.

Inclusion criteria	Exclusion criteria
• Studies investigating GD2 as a therapeutic target in bone sarcomas • Clinical and preclinical studies • Any publication of original data (clinical trial, retrospective trial, real world data) • Time of publication: January 2000–7 January 2026 • English language	• Tumors other than bone sarcoma • Studies investigating GD2 as diagnostic biomarker (imaging/diagnostic studies) • Systematic Reviews (paper without original data)

After duplicate removal and exclusion of articles published before 2000, titles and abstracts were screened to identify possible eligible articles and to exclude records that were clearly outside the scope of this review, including studies addressing unrelated topics or not reporting original data. The preliminary selection was further collegially discussed and validated by all reviewers. To maximize the completeness of the literature research, a supplementary manual search (snowballing approach) was also performed by screening the reference lists of all included articles to identify any additional relevant studies that met the predefined eligibility criteria.

Full-text articles of the remaining studies were independently assessed for eligibility according to the predefined inclusion and exclusion criteria.

The methodological quality of the selected studies was independently assessed by two reviewers (E.T., S.P.L.), using validated tools selected according to the study design described in the article as illustrated in **Supplementary Tables 2** and **3**. Conflicts between the two reviewers were solved by a third independent reviewer (S.D.A). Specifically, SYRCLE Risk of Bias tool was used for *in-vivo* animal studies (8), Cochrane Risk of Bias 2 (RoB 2) tool for randomized controlled trials (9) and ROBINS-I (10) was used for comparative non-randomized studies. ROBINS-I was also applied to early-phase, single-arm intervention studies to provide a consistent framework for risk-of-bias assessment across the clinical evidence reported. Clinical case reports or case series were analyzed using the Joanna Briggs Institute (JBI) Critical Appraisal Checklists (11). The risk-of-bias assessment for preclinical *in-vitro* studies was not evaluated, due to the lack of an appropriate tool for this type of articles identified in this literature research.

In addition, a search for ongoing clinical trials was performed using ClinicalTrials.gov (https://clinicaltrials.gov/) by a separate reviewer (S.B.) and the results were independently verified by two additional reviewers (E.T., S.P.L.). The search for clinical trials has been updated up to May 2026.

The following search terms were used, alone or in combination: “*GD2*”, “*anti-GD2*”, “*anti-GD2 antibody*”, “*GD2 antibody*”, “*sarcoma*”, “*bone sarcoma*”, “*Osteosarcoma*”, “*Ewing sarcoma*”, “*solid tumor*” and “*cancer*”. Eligible trials included studies specifically enrolling patients with OS, ES, or other primary bone sarcomas. Basket trials enrolling patients with GD2-positive solid tumors or sarcomas were also included when patients with primary bone sarcomas could be enrolled according to the eligibility criteria. Trials were excluded if they did not evaluate a GD2-directed therapeutic strategy or if patients with primary bone sarcomas were not eligible. Only trials listed as recruiting, not yet recruiting, or active but not recruiting were considered.

For studies selected according to the inclusion and exclusion criteria previously described, key variables were collected, including study type (prospective/retrospective, multi/single center, *in vivo*/*in vitro*), total number of patients, age range, experimental models used for preclinical studies, treatment modality (type of therapy, monotherapy/association, administration schedule), outcome measures used, point estimate with 95% confidence interval (CI) and p value.

## Results

**Figure 1:**
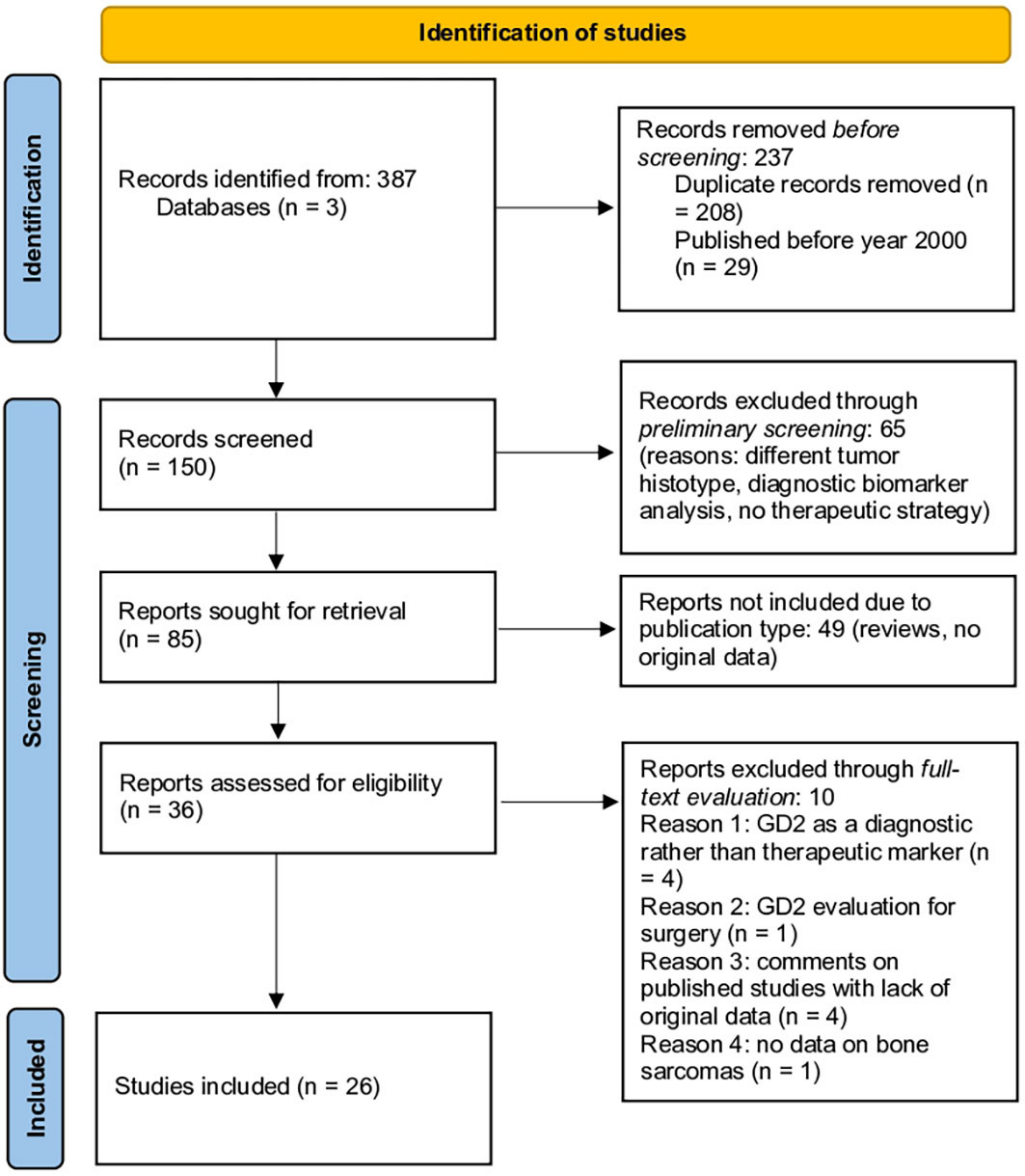
The PRISMA flow diagram visually represents the literature selection process.

The literature search of PubMed, Embase, and Web of Science identified a total of 387 records (**Figure 1**). After removal of duplicates and studies published before 2000, 150 articles remained for screening. Preliminary screening through evaluation of titles and abstracts resulted in the exclusion of 65 records that were considered outside the scope of the review as considering only tumor histotypes other than bone sarcomas, focusing on GD2 as a diagnostic biomarker, or not investigating any anti-GD2 therapeutic strategy. Additionally, 49 review articles were excluded as not reporting original data. Subsequently, 36 full-text articles were assessed for eligibility, leading to the exclusion of an additional 10 studies. Reasons for exclusion included investigation on GD2 as a diagnostic or surgical biomarker, absence of data on bone sarcomas or lack of original data.

Overall, 26 studies met the eligibility criteria and were included in this review (**Figure 1**). In addition, 15 registered clinical trials were identified according to the methodology described above.

### Literature review

Among the 26 included articles, 17 of them (65%) reported preclinical data, while 9 of them (35%) presented clinical data (**Tables 2**, **3**).

**Table 2:** List of preclinical studies on the role of GD2 in bone sarcomas included in the review.

First author	Year	Disease	Experimental model	Primary treatment tested	Monotherapy or association	Ref.
Kailayangiri	2012	ES	*In vitro*: ES cells; *in vivo*: ES murine xenografts	anti-GD2 CAR-T	Monotherapy	(43)
Liebsch	2013	ES	*In vivo*: ES murine patient derived xenografts	GD2-targeted T-cell	Monotherapy	(23)
Liu	2014	OS	*In vitro*: OS cell lines (Saos-2, MG-63, SJSA-1)	anti-GD2 mAb 14G2a	Association (Endothelin-1/Endothelin A receptor antagonist)	(16)
Fisher	2016	ES, NB	*In vitro*: commercial ES or NB cell lines; *in vivo*: ES and NB immunodeficient mouse models	anti-GD2 antibody ch14.18/CHO with Vγ9Vδ2+ γδT cells	Association (Zoledronic acid, IL-2)	(44)
Kailayangiri	2017	ES	*In vitro*: ES cells; *in vivo*: ES murine models	GD2-specific CAR-engineered human NK cells	Monotherapy	(25)
Zhu	2018	OS	*In vitro*: OS cell lines (MG-63,Saos-2)	anti-GD2 mAb 14G2a	Association (Cisplatin)	(15)
Chulanetra	2020	OS	*In vitro*: OS cell lines (HOS,U2OS)	anti-GD2 CAR-T	Monotherapy and association (Doxorubicin)	(21)
Park	2020	OS	*In vitro*: OS cell lines; *in vivo*: OS murine patient derived xenografts	Anti-GD2 BsAbs and ex vivo GD2-BATs	Monotherapy and association (anti-PD-L1)	(20)
Charan	2020	ES	*In vitro*: ES cell lines (ES2, ES4); *in vivo*: orthotopic and metastatic mouse models	anti-GD2 CAR-T	Monotherapy and association (HGF receptor neutralizing antibody (AMG102))	(22)
Chu	2021	OS, NB, Glioblastoma (GBM)	*In vitro*: OS, NB and GBM commercial cell lines; *in vivo*: OS, GBM or, NB immunodeficient mice xenografts	mAb anti-GD2 (Dinutuximab)	Association: N-803 (IL-15 superagonist) + ex vivo expanded peripheral blood NK cells	(17)
Golinelli	2022	ES	*In vitro*: ES cell lines (TC71, A673); *in vivo*: murine metastatic ES models	anti-GD2 bi-functional CAR mesenchymal stromal cells (MSCs)	Monotherapy	(24)
Theruvath	2022	OS, NB, SCLC	*In vitro*: OS, NB and SCLC commercial cell lines; *in vivo*: murine models	mAb anti-GD2 (Dinutuximab)	Association (anti-CD47 (B6H12, murine IgG1 antibody))	(14)
Wenyao	2024	OS	*In vitro*: OS cell lines (MG63, U2OS); *in vivo*: OS immunodeficient mouse models	mAb anti-GD2	Association (Niraparib)	(18)
Liatsou	2024	OS	*In vivo*: murine OS xenografts and spontaneously canine OS	humanized anti-GD2 hu3F8 antibody radiolabeled with actinium-225 ([225Ac]Ac-DOTA-hu3F8)	Monotherapy	(12)
Pezzella	2024	OS, ES, RMS	*In vitro*: OS, ES and RMS commercial cell lines; *in vivo*: OS and RMS murine orthotopic models	iCasp9.2A.GD2.CAR-CD28.4-1BBζ (CAR.GD2) T-cells	Monotherapy and association (Tazemetostat)	(45)
Shan	2025	OS	*In vitro*: OS cell lines (U2OS, 143B); *in vivo*: OS mouse xenograft models	anti-GD2 ADC (Naxitamab conjugated to mertansine)	Association (Tazemetostat)	(13)
Frapolli	2025	ES	*In vitro*: ES cell line (TC-71); *in vivo*: ES mouse model xenograft	mAb anti-GD2 (Dinutuximab beta)	Monotherapy and association (Doxorubicin)	(19)

ES, Ewing’s Sarcoma; OS, Osteosarcoma; GD2, Disialoganglioside 2; mAb, Monoclonal Antibodies; CAR-T, Chimeric Antigen Receptor T-Cells; NK, Natural Killer Cells; NB, Neuroblastoma; GBM, glioblastoma; RMS, Rhabdomyosarcoma; SCLC, Small-cell lung cancer; PDL1, Programmed-Death Ligand 1; ADC, Antibody Drug Conjugate.

**Table 3:** List of clinical studies on the role of GD2 in bone sarcomas included in the review.

First author	Year	Multi/Single center	Data collected prospectively/ retrospectively	Population	N	Age span	Primary treatment	Endpoint	Results	CI	Additional information (if reported)	Other point estimates	Ref
Hingorani	2022	Multi center	Prospectively	Recurrent pulmonary OS in complete surgical remission with unknown GD2 expression	39	0-30 (median 15)	Dinutuximab (70mg/m2/cycle) with GM-CSF - up to 5 cycles of mAb	DCR at 12 months	11/39 - 28.2%	95% CI: 15–44.9%			(31)
Spasov	2022	Single center	Retrospectively	Newly diagnosed metastatic GD2-positive ES/Ewing-Like Sarcoma (ELS)	3 (2/1)	3-16	Chemotherapy (VIDE + VAI) + 5 cycles of Dinutiximab beta (10 mg/m2/d over 10 d) + surgery and irradiation of the primary tumor and metastases	CR	Achieved by 3/3 pts (100%) - CR still ongoing after 14–19 months (at time of paper submission: march 2022)				(26)
Spasov	2025	Single center	Retrospectively	Newly diagnosed metastatic GD2-positive ES or ELS	9	5-17	Chemotherapy + Dinutiximab beta (10 mg/m2/d over 10 days for 5 cycles)	OS length/Median TTP	1877 days/1811 days				(27)
				Newly diagnosed ES (17 localized, 1 metastatic GD2-negative, 2 metastatic GD2-positive)	20	0-18	Chemotherapy	OS length/Median TTP	1547 days/1261 days				
				Relapsed/refractory GD2-positive ES	4	1-18	Chemotherapy + Dinutiximab beta (10 mg/m2/d over 10 days for 5 cycles)	OS length/Median TTP	810 days/782 days				
				Relapsed/refractory metastatic ES	4	9-18	Chemotherapy	OS length/Median TTP	210 days/113 days				
Wingerter	2021	Single center (Case report)	Retrospectively	Primary refractory GD2-positive ES	1	5	Chemotherapy (irinotecan/temozolomide - IT) and Dinutuximab beta (100 mg/m²/10 days; continuous intravenous infusion)	PFS length	12 months				(33)
Rosenbaum	2021	Multi center	Prospectively	Metastatic or distant relapsed sarcoma	135 (68/68) - 14 OS patients (no other details are reported)	>16 years - mean 51.1	KLH-conjugated ganglioside vaccine targeting GM2, GD2 and GD3 + OPT-821 vs OPT-821 alone	RFS at 12 months	34.5% vs 34.8%*; p = 0.725; median RFS 191 days in both arms	CI: 104–420 in the vaccine arm and 141–357 in the control			(35)
								1-year Overall Survival and serologic response	1-year Overall Survival: 93.1% vs 91.5% (p = 0.578). Serologic response at last time point (weeks 40 – 68; any anti-ganglioside antibody titer of > 1:20): 89.3% on vaccine arm vs. 20.8% on the adjuvant only.				
Kaczanowska	2024	Single center	Prospectively	OS/NB	11/2	8–28 (median 17)	GD2 CAR-Ts at the four planned dose levels (1 × 105, 1 × 106, 3 × 106, and 1 × 107 transduced GD2 CAR-Ts/kg)	Toxicity	15.4% (2/13) grade-1 cytokine release syndrome (CRS). No dose-limiting toxicities. No need of iCasp9 suicide gene switch.				(34)
Yankelevich	2024	Multi center	Prospectively	NB/OS/Desmoplastic small round cell tumor	5/3/1	<30	GD2 BATs: 3 + 3 dose escalation design with dose levels of 40, 80, and 160×106 GD2 BATs/kg/infusion twice per week + Low-dose IL-2 (300,000 IU/m2/day) and biweekly subcutaneous GM-CSF (250 µg/m2/dose).	Toxicity	All mild CRS but dose-limited-toxicities; maximum-tolerated dose (MTD) was not reached.		Efficacy information reported: no objective responses according to INRC or RECIST. 6/9 (3/5 NB, 2/3 OS, 1/1 DSRCT) had PD, and 3 patients (2/5 NB, 1/3 OS) had SD. 5/9 (1 OS, 4 NB) survived 1-year post-GD2BAT therapy. The median overall survival was 18 months		(28)
Bishop	2020	Single center	Prospectively	R/R OS	6	12-21	Hu14.18K322A - humanized anti-GD2 mAb (60 mg/m2/dose) daily schedule (over 4 h/day for 4 days every 28 days (1 course); total of 14 courses (median 2, range 1-6)	Toxicity	Similar; Limited ≥grade 3 toxicities other than pain in both schedules; weekly schedule fewer grade 1–2 fatigue, hypertension, respiratory symptoms, and hypoxia.		Efficacy information reported: Daily schedule: 3 SD as best response, 5/6 PD while on study; weekly schedule: 2 SD and 3 PD as best overall response.		(29)
				R/R OS/NB	2/3	4-16	Weekly schedule: hu14.18K322A (40 mg/m2/dose) over 4 h/once a week for 4 weeks (1 course); total of 7 courses (median 1, range 1-3)	Pharmacokinetics	Both schedules maintained concentrations over the 4 week-period above 1 ng/mL (threshold level for detection of ADCC in preclinical studies of hu14.18K322A).				
Varo	2023	Single center	Prospectively	NB/Retinoblastoma (RB)/OS	17/1/0		Standard Infusion Regimen (SIR): Naxitamab 60-min infusion of 3 mg/kg/day on Day 1 of cycle 1 and a 30- to 60-min infusion on Day 3 and Day 5.	G3 Adverse Events (AE)	G3 AE was reduced from 8.1% with SIR to 2.5% with STU. The odds of an infusion being associated with a G3 AE reduced by 70.3% with STU vs. SIR (odds ratio: 0.297; p = 0.037).	The comparable pharmacokinetics of Naxitamab during SIR and STU may indicate that switching to STU reduces G3 AEs without impact on efficacy.			(30)
				NB/RB/OS	10/0/1		Step-UP Regimen (STU): Naxitamab 2-h infusion on Day 1, initiated at a rate of 0.06 mg/kg/h during 15 min (0.015 mg/kg), increased gradually to a cumulative dose of 3 mg/kg; on Days 3 and 5, the 3-mg/kg dose is initiated at 0.24 mg/kg/h (0.06 mg/kg) and delivered in 90 min according to the same gradual-increase strategy.	Pharmacokinetics	Mean serum Naxitamab levels pre- and post-STU (11.46 µg/ml pre-infusion; 100.95 µg/ml post-infusion) were within the range reported for SIR.				

* the results here reported refer to the overall population, no specific data for OS patients are reported.

ES, Ewing’s Sarcoma; OS, Osteosarcoma; ELS, Ewing-Like Sarcoma; GD2, Disialoganglioside 2; mAb, Monoclonal Antibodies; DCR, Disease Control Rate; CR, Complete Remission; pts, patients; VIDE, Vincristine, Ifosfamide, Doxorubicin, Etoposide; VAI, Vincristine, Dactinomycin, Ifosmanide; OS length, Overall Survival length; TTP, Time To Progression; PFS, Progression-Free Survival; RFS, Relapse-Free Survival; CAR-T, Chimeric Antigen Receptor T-Cells; CRS, Cytokine Release Syndrome; AE, Adverse Events; G3, Grade 3; R/Rm Recurrent/Refractory; NBm Neuroblastoma; RB, retinoblastoma; SIR, Standard Infusion Regimen; STU, STep-Up; ADC: Antibody Drug Conjugate.

Preclinical studies predominantly used OS models (n=9), followed by ES models (n=7), with one study including both tumor types. Three studies were limited to *in vitro* experiments using OS cell lines, two were exclusively *in vivo*, whereas the majority (n=12) combined both experimental approaches. *In vivo* studies consistently utilized murine xenograft models of OS or ES, with only one study extending the analysis to a spontaneous canine OS model (12).

The therapeutic approaches explored across these studies included humanized anti-GD2 monoclonal antibodies (mAbs) such as Dinutuximab, Dinutuximab beta and Naxitamab, administered either as monotherapy or in association with chemotherapy or other strategies (13–19).

Overall, anti-GD2 mAbs bind to GD2 on tumor cells and engage immune effector cells, such as Natural Killer (NK) cells and macrophages, triggering antibody-dependent cellular cytotoxicity (ADCC) and complement-mediated tumor cell lysis (19).

The treatment combinations published in literature were effective in reducing tumor growth and metastatic spread through increased expression of apoptosis-related genes and pro-apoptotic factors (13, 14), activation of endoplasmic reticulum (ER)-stress-associated apoptotic pathways (15) and inhibition of cell invasiveness (16). In ES *in vitro* models, Dinutuximab Beta induced a rapid initial response, likely driven by early complement activation; however, its efficacy declined over time (19). Yet, combination with chemotherapy, such as Doxorubicin, overcomes this limitation, enhancing antitumor activity and resulting in reduced tumor progression and prolonged survival (19).

Noteworthy, one study focused on radio-immunotherapy with humanized anti-GD2 hu3F8 antibodies radiolabeled with actinium-225 ([225Ac]Ac-DOTA-hu3F8) showing efficacy in delaying tumor growth in murine models and progression of metastatic spread and overall survival in spontaneously occurring canine OS (12).

Furthermore, advanced immunotherapeutic approaches were also investigated, such as anti-GD2 bispecific antibodies-armed T cells (GD2-BATs) which demonstrated a high anti-tumor activity against OS both *in vitro* and *in vivo* and even enhanced efficacy when combined with anti-PDL1 therapies (20).

Studies on GD2 chimeric antigen receptor (CAR) T-cells showed promising results in delaying tumor growth in OS and ES cells and in reducing metastasis in murine ES models (21–23). Two additional studies investigated GD2-specific CAR-engineered human Mesenchymal Stem Cells (MSCs) and NK cells, demonstrating only partial antitumor activity (24, 25).

Regarding clinical studies published in literature (n=9), six of them enrolled patients affected by OS while three by ES. Overall, studied populations were heterogeneous in terms of age range, disease stage and anti-GD2 therapeutic approaches.

Most studies included patients with relapsed or refractory disease, except for two studies conducted by the same group, which enrolled patients with newly diagnosed metastatic ES (26, 27).

Investigated anti-GD2 therapeutic strategies were mAbs such as Dinutuximab, Dinutuximab beta, and Naxitamab, administered either as monotherapy or in combination regimens, anti-CD3×anti-GD2 BATs, a trivalent ganglioside vaccine targeting GD2, GM2, and GD3, and GD2-directed CAR-T cell therapies.

The clinical studies published in literature were widely heterogeneous also in terms of primary objectives and endpoints analyzed. Indeed, six studies focused on treatment efficacy as primary endpoint, using different outcome measures, while three studies mainly focused on safety of anti-GD2 treatments, comparing different therapeutic schedules and infusion regimens (28–30).

Results for clinical studies on mAbs concerned both OS and ES patients. One study assessed the efficacy of Dinutuximab with Granulocyte-Macrophage Colony stimulating factor (GM-CSF) in 39 patients with recurrent pulmonary OS with unknown GD2 tumor expression. The study reported a higher 12-month disease control rate (DCR) compared with the historical benchmark (28.2% vs 20%); however, the predefined efficacy threshold was not achieved, as only 11 of 39 patients remained event-free compared with the prespecified cutoff of 16 of 39 patients (31).

Notably, Spasov et al. investigated the addition of Dinutuximab beta to standard chemotherapy (according to the EURO EWING 2012 Protocol (32)) and surgery in patients with metastatic ES, both at diagnosis and at relapse. This approach showed an improved overall survival and prolonged median time to progress (TTP) compared with matched controls treated with chemotherapy and surgery alone (26, 27). Consistently, a separate study described a single clinical report of a patient with refractory ES treated with Dinutuximab beta in combination with chemotherapy (irinotecan and temozolomide) after surgery and radiotherapy (33). The patient achieved disease control for approximately 12 months (33). FDG-PET imaging performed 26 weeks after surgery revealed no evidence of active disease and overall, progression free survival was approximately 12 months (33).

Regarding GD2 CAR-T cell therapy, its use was evaluated by Kaczanowska et al. in a cohort of 11 OS and 2 NB patients (34). Treatment was well tolerated, with no significant toxicities reported and no patients required administration of the iCasp9 suicide gene switch for unacceptable toxicity related to GD2 CAR-Ts (34). Although all patients ultimately experienced disease progression, three patients with a robust CAR-T cell expansion achieved prolonged stable disease beyond day 90, underscoring the need to further elucidate the determinants of CAR-T expansion in solid tumors to improve therapeutic efficacy (34).

Furthermore, a trivalent ganglioside vaccine targeting GD2, GM2 and GD3 was evaluated in a cohort of 136 patients with metastatic or relapsed sarcomas, including a small cohort of patients with OS (10%) (35). Despite inducing a sustained serologic response, no significant improvement in recurrence-free or overall survival was observed compared with patients receiving the immunologic adjuvant OPT-821 alone (35).

### Registered clinical trials

Fifteen registered trials have been identified on ClinicalTrials.gov: 9 actively recruiting, 2 not yet recruiting and 4 with completed recruitments (**Table 4**).

**Table 4:** List of registered clinical trials on the role of GD2 in bone sarcomas included in the review.

NCT identifier	Official title	Phase	Disease	Age	Primary purpose/ study type	Treatment	Interventional model	Outcome	Masking	Enrollment (estimated number)	Recruitment status
NCT01953900	Vaccination to Enhance the Anti-Tumor Activity of GD2 Chimeric Antigen Receptor-Expressing, VZV-Specific T Cells in Subjects With Advanced Sarcomas and Neuroblastoma (VEGAS)	Phase I	R/R OS or R/R HR-NB not responsive to standard treatment	All ages	Treatment/ Interventional	autologous iC9-GD2-CAR-VZV-CTLs in combination with VZV vaccination	Single Group Assignment	Primary outcome: Number of patients with dose limiting toxicity. Secondary outcome: Amount of T cells in the blood after the infusions, Number of patients with a response to the T cells	None (Open Label)	26	Completed recruitment
NCT05400603	A Phase I Study of Allogeneic Ex Vivo Expanded Gamma Delta (γδ) T Cells in Combination With Dinutuximab, Temozolomide, Irinotecan, and Zoledronate in Children With Refractory/Relapsed, or Progressive Neuroblastoma or Refractory/Relapsed Osteosarcoma	Phase I	R/R OS and HR-NB	≥12 months	Treatment/ Interventional	Ex Vivo Expanded Allogeneic γδ T Cells in Combination with Dinutuximab, Temozolomide, Irinotecan and Zoledronate	Sequential Assignment	Determine the maximum tolerated dose and recommended Phase II dose	None (Open Label)	24	Active recruitment
NCT05130255	Phase 1 Trial With GD2-SADA:177Lu-DOTA Drug Complex in Patients With Recurrent or Refractory Metastatic Solid Tumors Known to Express GD2, Including Small Cell Lung Cancer, High Risk Neuroblastoma, Sarcoma and Malignant Melanoma	Phase I	R/R metastatic GD2-positive solid tumors	≥16 years	Treatment/ Interventional	GD2-SADA:177Lu-DOTA Complex	Single Group Assignment - A phase 1 dose-escalation single-arm, open-label, non-randomized, multi-center trial.	Primary outcomes: To determine the optimal, safe GD2-SADA protein dose and dosing interval between GD2-SADA and 177Lu-DOTA administrations. To determine maximum tolerable activity of 177Lu-DOTA. To assess cumulative toxicity signals and safety profile.	None (Open Label)	60	Active recruitment
NCT03721068	A Phase I Study of Autologous Activated T-Cells Expressing a 2nd Generation GD2 Chimeric Antigen Receptor, IL-15, and iCaspase9 Safety Switch Administered To Patients With Relapsed/Refractory Neuroblastoma or Relapsed/Refractory Osteosarcoma	Phase I	R/R NB or OS	≥18 months	Treatment/ Interventional	iC9.GD2.CAR.IL-15 T cells	Single Group Assignment	To identify the maximum tolerated dose and to evaluate the expansion and persistence of iC9.GD2.CAR.IL-15 cells *in vivo*. To determine the anti-tumor response rate, Overall Survival, PFS.	None (Open Label)	18	Active recruitment
NCT06839703	International Open-label Phase I Dose Escalation Study of Dinutuximab Beta in Combination With Vincristine/Doxorubicin/Cyclophosphamide and Ifosfamide/Etoposide in Pediatric, Adolescent, and Adult Patients With GD2-positive Ewing sarcoma	Phase I	ES at diagnosis	≥12 months	Treatment/ Interventional	Dinutuximab beta	Single Group Assignment	To investigate the feasibility, toxicity, and biological activity of Dinutuximab beta in combination with standard chemotherapy in ES.	None (Open Label)	18	Not yet recruiting
NCT07211737	(INCITE-ON) Phase I Study of i15.NKG2D.Zeta-NK Cell Conditioning in the Tumor Micro-environment in Combination With C7R.GD2.CAR-T for the Treatment of Patients With Relapsed or Refractory Osteosarcoma or Neuroblastoma	Phase I	R/R NB and OS	1–24 years	Treatment/ Interventional	i15.NKG2D.zeta NK cells and C7R.GD2.CAR-T cells	Single Group Assignment	Primary outcomes: Dose-limiting toxicity rate, and Maximum Tolerated Dose. Secondary outcomes: ORR, Cytokine release syndrome grade	None (Open Label)	27	Not yet recruiting
NCT01953900	Vaccination to Enhance the Anti-Tumor Activity of GD2 Chimeric Antigen Receptor-Expressing, VZV-Specific T Cells in Subjects With Advanced Sarcomas and Neuroblastoma (VEGAS)	Phase I	R/R OS or R/R HR-NB not responsive to standard treatment	All ages	Treatment/ Interventional	autologous iC9-GD2-CAR-VZV-CTLs in combination with VZV vaccination	Single Group Assignment	Primary outcome: Number of patients with dose limiting toxicity. Secondary outcome: Amount of T cells in the blood after the infusions, Number of patients with a response to the T cells	None (Open Label)	26	Completed recruitment
NCT03635632	Phase I Study of Autologous T Lymphocytes Expressing GD2-specific Chimeric Antigen and Constitutively Active IL-7 Receptors for the Treatment of Patients With Relapsed or Refractory Neuroblastoma and Other GD2 Positive Solid Cancers (GAIL-N)	Phase I	R/R NB or OS and other GD2-positive solid tumors	1–74 years	Treatment/ Interventional	C7R-GD2.CAR-T cells	Single Group Assignment	Primary outcome: Determine maximum tolerated dose. Secondary: Determine Anti-tumor Responses	None (Open Label)	94	Completed recruitment
NCT02992210	Multicenter Trial of Phase I/II Studies on GD2 Positive Solid Tumors by 4SCAR-GD2	Phase I/II	GD2-positive tumors R/R, non-resectable or metastatic after standard first-line therapy	1–65 years	Treatment/ Interventional	4SCAR-GD2 T cells	Single Group Assignment	Primary: Number of patients with adverse events. Secondary: Anti-tumor effects and the survival time of the patients	None (Open Label)	100	Active recruitment
NCT05438368	GD2/CD70 Bi-specific CAR-T Cells for Cancer Treatment	Phase I/II	GD2 and/or CD70 positive tumors	1–75 years (body weight ≥10 kg)	Treatment/ Interventional	Infusion of bi-4SCAR- GD2/CD70 T cells at 10^6 cells/kg body weight via IV	Single Group Assignment	To assess the feasibility, safety and efficacy of the treatment. To evaluate the function of the GD2/CD70 bi-specific CAR-T cells and their persistence.	None (Open Label)	30	Active recruitment
NCT05437328	GD2/CD56 Bi-specific CAR-T Cells for Cancer Treatment	Phase I/II	GD2 and/or CD56 positive tumors	1–75 years (body weight ≥10 kg)	Treatment/ Interventional	Infusion of bi-4SCAR- GD2/CD56 T cells at 10^6 cells/kg body weight via IV	Single Group Assignment	To evaluate the feasibility, safety and efficacy of the treatment.	None (Open Label)	60	Active recruitment
NCT05437315	GD2/PSMA Bi-specific CAR-T Cells For the Treatment of GD2 and PSMA Positive Solid Tumors	Phase I/II	GD2 and/or PSMA positive tumors	1–75 years (body weight ≥10 kg)	Treatment/ Interventional	Infusion of bi-4SCAR- GD2/PSMA T cells at 10^6 cells/kg body weight via IV	Single Group Assignment	To determine the toxicity profile.	None (Open Label)	60	Active recruitment
NCT03373097	Phase I/II Study of Anti-GD2 Chimeric Antigen Receptor-Expressing T Cells in Pediatric Patients Affected by High Risk and/or Relapsed/Refractory Neuroblastoma or Other GD2-positive Solid Tumors	Phase I/II	R/R NB (Phase I/II) and other GD2-positive tumors considered incurable with conventional treatments by the treating physician (Phase II only)	1–25 years	Treatment/ Interventional	GD2-CAR-T01	Single Group Assignment	Phase I - Identification of the dose limiting toxicity. Phase II - Antitumor effect.	None (Open Label)	42	Completed recruitment
NCT05968768	To Evaluate the Efficacy and Safety of Naxitamab in Patients With Refractory Ewing’s Sarcoma	Phase II	Refractory ES	2–21 years	Treatment/ Interventional	Naxitamab	Parallel Assignment	Primary: Safety assessment. Secondary: EFS, PFS, ORR, Overall Survival	None (Open Label)	24	Active recruitment
NCT02502786	A Phase II Study of Humanized Monoclonal Antibody 3F8 (Hu3F8) With Granulocyte-Macrophage Colony Stimulating Factor (GM-CSF) in the Treatment of Recurrent Osteosarcoma	Phase II	Recurrent OS	13 months-40 years	Treatment/ Interventional	humanized anti-GD2 antibody	Single Group Assignment	EFS, Time to recurrence	None (Open Label)	46	Completed recruitment
NCT06669013	A Randomized, Multicenter, Open-label Phase III Study of Dinutuximab Beta With Investigator’s Choice of Chemotherapy in Patients Under 18 Years of Age With Bone and Soft Tissue Sarcomas With High Levels of GD2 Expression and Disease Progression During 1st Line Chemotherapy	Phase III	Advanced/ metastatic GD2-positive positive ES, OS and RMS after progression on 1st line of chemotherapy	≤ 18 years	Treatment/ Interventional	Chemotherapy + Immunotherapy (Dinutuximab beta)	Parallel Assignment	Primary outcome: PFS, Secondary outcome: Overall Survival, ORR, TCR, DR, IAAE	Single (Participant)	40	Active recruitment

ES, Ewing’s Sarcoma; OS, Osteosarcoma; RMS, Rhabdomyosarcoma; GD2, Disialoganglioside 2; PFS, Progression-Free Survival; ORR, Overall response rate; TCR, Tumor control rate; DR, Duration of response; IAAE, Immune-associated adverse events; NB, Neuroblastoma; HR, High Risk; mAb: Monoclonal Antibodies; DCR, Disease Control Rate; CR, Complete Remission; EFS, Event Free Survival; R/R, Recurrent/Refractory; CAR-T, Chimeric Antigen Receptor T-Cells; VZV, Varicella-Zoster Virus.

Five trials are designed to be focused on mAbs, specifically one trial on Dinutuximab (NCT05400603), two trials on Dinutuximab Beta (NCT06669013, NCT06839703), one on Naxitamab (NCT05968768) and one on Humanized Monoclonal Antibody 3F8 (Hu3F8) mAb (NCT02502786). All mAbs were planned to be administered in combination with standard chemotherapy regimens, with only Hu3F8-mAb being administered with GM-CSF alone.

Nine trials involved the use of GD2-CAR-Ts and one of radio-immunotherapy with GD2-SADA:177Lu-DOTA Complex (NCT05130255).

Concerning tumor types, 6 trials were planned to enroll patients with OS, patients with ES in 2 trials, and patients with GD2-positive solid tumors in general in 7 trials. All studies involved high-risk refractory/relapsed or progressive tumors with only one study enrolling high-risk ES patients at diagnosis to investigate feasibility, toxicity, and biological activity of treatment with Dinutuximab Beta in combination with standard chemotherapy. No studies on anti-GD2 treatments in newly diagnosed OS patients are currently ongoing.

The majority of the identified trials are phase I or phase I/II, with only one being a phase III trial, specifically designed to investigate the use of Dinutuximab Beta with chemotherapy in patients with GD2-positive ES, OS or rhabdomyosarcoma with disease progression after 1^st^ line chemotherapy (NCT06669013).

## Discussion

Primary bone sarcomas, such as OS and ES, are rare malignancies with a poor prognosis, especially in patients presenting with metastatic or relapsed disease, for whom survival outcomes remained largely unchanged over the past decades (1–3). This highlights a critical unmet need for development of novel, disease-specific therapeutic strategies capable of improving patient outcomes, particularly in the pediatric population, where treatment resistance and side effects of conventional therapies remain significant challenges (1–3).

In this context, Disialoganglioside GD2 emerges as a promising therapeutic candidate, due to its high expression in several pediatric malignancies, including OS and ES, and its involvement in key oncogenic processes of tumor progression and treatment resistance (4, 5). Notably, GD2 expression appears to be maintained from diagnosis through relapse, further supporting its relevance as a stable and actionable target (6).

In pediatric oncology, GD2 already represents a well-established and clinically relevant target in the treatment of NB, with the success of GD2-directed mAbs in high-risk NB strongly supporting its investigation in other GD2-positive pediatric tumors.

Dinutuximab Beta is currently considered the standard of care for maintenance therapy of high-risk NB, both in frontline and relapsed/refractory settings, with significant improvements in survival outcomes (36). Moreover, recent pediatric clinical trials as COG ANBL1221 (37) and BEACON-Immuno (38), demonstrate increased efficacy of the combination of chemotherapy and immunotherapy with anti-GD2 mAb (chemo-immunotherapy) in relapsed/refractory NB. Chemo-immunotherapy strategy with anti-GD2 mAb improved drastically the outcome of patients with relapsed/refractory NB, reaching an objective response rate of approximately 40% (36). Its use in this clinical setting is increasingly being incorporated into clinical practice in pediatric oncology expert Centers (36, 39, 40).

Starting from the success of the anti-GD2 strategies in NB, the therapeutic landscape is rapidly evolving, with the development of next-generation GD2-targeted approaches, including novel mAbs such as Naxitamab, as well as cellular therapies such as GD2-specific CAR-T cells, reflecting growing interest in this field (41).

Through this systematic review, we provide a comprehensive overview of preclinical and clinical evidence on anti-GD2 strategies in pediatric bone sarcomas. Overall, available data support a strong biological rationale for GD2 targeting; however, several limitations and challenges remain in clinical translation, highlighting the urgent need to implement further research in this field.

Preclinical data consistently demonstrate the antitumor activity of GD2-directed approaches. MAbs, in association with chemotherapy or other agents, show enhanced efficacy in bone sarcoma models through multiple mechanisms, including induction of apoptosis, activation of ER-stress pathways, and inhibition of tumor invasiveness (13–16). The synergistic effect between anti-GD2 mAbs and chemotherapy may reflect the ability of chemotherapy to overcome resistance and sustain immune-mediated tumor killing (19). Histopathological analyses performed on *in-vitro* ES models treated with Dinutuximab beta and Doxorubicin, support this interaction, showing that while all treatments induce tumor necrosis, Doxorubicin also promotes apoptosis, potentially contributing to the improved efficacy observed with chemo-immunotherapy (19).

Similarly, GD2BATs exhibit potent anti-tumor activity, further enhanced if combined with immune checkpoint inhibitors (20). Cell-based therapies, such as GD2-specific CAR-Ts, also show promising activity in preclinical OS and ES models, reducing tumor growth and metastatic dissemination (21–23). Further emerging strategies such as radio-immunotherapy demonstrate encouraging preclinical results, warranting further clinical evaluation (12).

Overall, these findings provide promising preliminary preclinical evidence of the efficacy of GD2-directed therapies, further enhanced when integrated into multimodal treatment approaches, likely through synergistic effects on bone sarcoma tumor cells and microenvironment.

Next, we identify ongoing and recently completed clinical trials to explore the available clinical evidence on the use of anti-GD2 strategies in pediatric bone sarcomas.

Although still limited and heterogeneous, clinical data provide early evidence on the feasibility and safety of anti-GD2-based approaches in this field. Currently, however, most published studies include small patient cohorts, retrospective single-center experiences, or individual case reports, supporting a preliminary rationale but precluding robust conclusions on definite survival benefit or treatment effectiveness in pediatric bone sarcomas.

MAbs such as Dinutuximab and Dinutuximab beta, particularly when combined with chemotherapy and/or immunostimulatory agents, demonstrate encouraging results in terms of disease control and survival outcomes, especially in ES patients both in relapsed/refractory settings and in newly diagnosed high-risk disease (26, 27). However, as these observations derive from retrospective, single-center analysis, and isolated case reports, they should be interpreted with caution, while validation in prospective, multicenter studies remains an unmet need.

Regarding relapsed OS, Hingorani et al. conducted one of the largest clinical studies investigating Dinutuximab in combination with GM-CSF in 39 patients (31). Although the study reported a higher 12-month DCR compared with historical benchmarks, the primary endpoint was not met, thus not providing consistent evidence for routine clinical use of anti-GD2 mAbs in OS (31). One limitation of the study was however the lack of evaluation of tumor GD2 expression, probably preventing appropriate patient stratification according to target expression, suggesting the need for further investigation in prospective clinical trials for OS patients (31).

Beyond mAbs, other therapeutic strategies have been developed and evaluated in clinical settings. GD2 CAR-Ts, show favorable safety profiles but only limited clinical efficacy, with biological barriers as insufficient CAR-T expansion and persistence, tumor heterogeneity and interaction with tumor microenvironment still limiting the use of adoptive cell therapy in solid tumors. Further investigations are needed to understand drivers and determinants of CAR-Ts expansion in heavy pre-treatment patients with solid tumors, in order to achieve longer disease control and to improve therapeutic effectiveness also in bone sarcomas (34).

Similarly, vaccine-based strategies targeting GD2 and other gangliosides induce sustained immunogenic responses, but fail to translate into significant clinical benefit (35).

The analysis of registered clinical trials further highlights a growing interest in GD2-directed therapies. Current studies are early-phase, primarily focus on safety and biological activity of the investigated strategies and mainly enroll patients with relapsed/refractory sarcomas. The results of these studies are expected to inform the design of subsequent phase III trials and to support the development and potential earlier integration of anti-GD2 strategies into the management of high-risk pediatric bone sarcoma patients, as successfully demonstrated in NB (36, 39, 40).

Despite these encouraging findings, several limitations must be acknowledged. The translation from preclinical models into clinical benefit in a pediatric setting remains challenging and the available clinical evidence is limited by small sample sizes, lack of randomized controlled trials, heterogeneity in study design and variability in outcome measures, thereby strongly limiting the ability to draw definitive conclusions regarding treatment efficacy.

A key translational challenge lies in the identification of patients most likely to benefit from GD2-directed therapies. Currently, the lack of standardized methodologies for assessing GD2 expression represents a major limitation (4). Multiple assays have been developed to evaluate GD2 expression in tumor tissues, including immunofluorescence, flow cytometry, and mass spectrometry; however, these approaches are not standardized, and clinically relevant thresholds of GD2 expression have not been clearly defined. This variability hampers the use of GD2 as a predictive biomarker, limits appropriate patient selection for clinical trials, and complicates the comparison of results across studies. Therefore, the development of robust, standardized, and clinically validated assays to quantify GD2 expression is urgently needed to enable better patient stratification and to guide the design of future trials, including earlier-phase studies (4). In addition, complementary approaches such as circulating GD2 measurements (42) may further support biomarker development and improve the identification of patients most likely to benefit from GD2-targeted therapies.

The aim of this review was to provide the broadest possible overview of the available evidence on this topic. However, the identified preclinical and clinical studies were highly heterogeneous in terms of study design, experimental models, investigated drugs, and reported outcomes. To improve transparency, we also performed a risk of bias assessment of the included studies. Nevertheless, no validated risk of bias tool is currently available for some study designs, such as *in vitro* preclinical studies and single-arm clinical trials. Given the rarity of these diseases, the methodological limitations identified were expected.

Finally, the available evidence identifies GD2 as a biologically relevant therapeutic target in pediatric bone sarcomas, with consistent preclinical data demonstrating antitumor activity across multiple therapeutic modalities. However, despite initial promising results especially of combination strategies, available clinical evidence remains preliminary and nowadays there are no data to demonstrate a clear survival benefit of GD2-directed therapies in routine clinical practice.

At present, these approaches should therefore be regarded as investigational, with a strong need of implementation in well-designed prospective clinical trials. In this context, the results of the randomized ongoing trial NCT06669013 are expected to add new clinical information soon.

Given the rarity and biological complexity of these tumors, collaborative efforts are essential to advance research in this field. Establishing cooperative groups and international consortia is therefore crucial to harmonize study designs, facilitate patient enrollment in early-phase trials and accelerate the development and validation of novel therapeutic strategies, ultimately improving clinical outcomes for patients with rare malignancies.

## Data Availability

The original contributions presented in the study are included in the article/(**Supplementary Material**. Further inquiries can be directed to the corresponding author.
