# Supplementary material for: GD2-targeted immunotherapy in pediatric bone sarcomas: a systematic review of emerging strategies and combination approaches
Source: Front Immunol. 2026 Aug 4;17:1895000. doi: 10.3389/fimmu.2026.1895000 (PMC13481727; doi:10.3389/fimmu.2026.1895000)
Supplement: Supplementary file 1 [file Table1.docx]

**Supplementary Table 1 - List of literature search strings**

| **Database 1**: PubMed  **Coverage**: Database inception to 7 January 2026  **Search date**: 7 January 2026 | | |
| --- | --- | --- |
| **Search Number** | **Query** | **Search Details** |
| 1 | Bone sarcomas and GD2 | "(""osteosarcoma""[MeSH Terms] OR ""osteosarcoma""[All Fields] OR (""bone""[All Fields] AND ""sarcomas""[All Fields]) OR ""bone sarcomas""[All Fields]) AND ""gd2""[All Fields]" |
| 2 | Osteosarcoma and GD2 | ("osteosarcoma"[MeSH Terms] OR "osteosarcoma"[All Fields] OR "osteosarcomas"[All Fields]) AND "gd2"[All Fields] |
| 3 | Ewing sarcoma and GD2 | ("sarcoma, ewing"[MeSH Terms] OR ("sarcoma"[All Fields] AND "ewing"[All Fields]) OR "ewing sarcoma"[All Fields] OR ("ewing"[All Fields] AND "sarcoma"[All Fields])) AND "gd2"[All Fields] |
| 4 | Pediatric sarcomas and GD2 | ("paediatrics"[All Fields] OR "pediatrics"[MeSH Terms] OR "pediatrics"[All Fields] OR "paediatric"[All Fields] OR "pediatric"[All Fields]) AND ("sarcoma"[MeSH Terms] OR "sarcoma"[All Fields] OR "sarcomas"[All Fields] OR "sarcoma s"[All Fields]) AND "gd2"[All Fields] |
| 5 | Pediatric sarcomas and anti-GD2 | "(""paediatrics""[All Fields] OR ""pediatrics""[MeSH Terms] OR ""pediatrics""[All Fields] OR ""paediatric""[All Fields] OR ""pediatric""[All Fields]) AND (""sarcoma""[MeSH Terms] OR ""sarcoma""[All Fields] OR ""sarcomas""[All Fields] OR ""sarcomas""[All Fields]) AND ""anti-GD2""[All Fields]" |
| 6 | Bone sarcomas and anti-GD2 | ("osteosarcoma"[MeSH Terms] OR "osteosarcoma"[All Fields] OR ("bone"[All Fields] AND "sarcomas"[All Fields]) OR "bone sarcomas"[All Fields]) AND "anti-GD2"[All Fields] |
| 7 | Ewing sarcoma and anti-GD2 | ("sarcoma, ewing"[MeSH Terms] OR ("sarcoma"[All Fields] AND "ewing"[All Fields]) OR "ewing sarcoma"[All Fields] OR ("ewing"[All Fields] AND "sarcoma"[All Fields])) AND "anti-GD2"[All Fields] |
| 8 | Osteosarcoma and anti-GD2 | "(""osteosarcoma""[MeSH Terms] OR ""osteosarcoma""[All Fields] OR ""osteosarcomas""[All Fields]) AND ""anti-GD2""[All Fields]" |
| 9 | Osteosarcoma and Disialoganglioside GD2 | ("osteosarcoma"[MeSH Terms] OR "osteosarcoma"[All Fields] OR "osteosarcomas"[All Fields]) AND ("disialoganglioside"[All Fields] OR "sialogangliosides"[Supplementary Concept] OR "sialogangliosides"[All Fields] OR "disialogangliosides"[All Fields]) AND "GD2"[All Fields] |
| 10 | Ewing sarcoma and Disialoganglioside GD2 | ("sarcoma, ewing"[MeSH Terms] OR ("sarcoma"[All Fields] AND "ewing"[All Fields]) OR "ewing sarcoma"[All Fields] OR ("ewing"[All Fields] AND "sarcoma"[All Fields])) AND ("disialoganglioside"[All Fields] OR "sialogangliosides"[Supplementary Concept] OR "sialogangliosides"[All Fields] OR "disialogangliosides"[All Fields]) AND "GD2"[All Fields] |
| 11 | Pediatric sarcomas and Disialoganglioside GD2 | "(""paediatrics""[All Fields] OR ""pediatrics""[MeSH Terms] OR ""pediatrics""[All Fields] OR ""paediatric""[All Fields] OR ""pediatric""[All Fields]) AND (""sarcoma""[MeSH Terms] OR ""sarcoma""[All Fields] OR ""sarcomas""[All Fields] OR ""sarcoma s""[All Fields]) AND (""disialoganglioside""[All Fields] OR ""sialogangliosides""[Supplementary Concept] OR ""sialogangliosides""[All Fields] OR ""disialogangliosides""[All Fields]) AND ""GD2""[All Fields]" |
| 12 | Bone sarcomas and Disialoganglioside GD2 | "(""osteosarcoma""[MeSH Terms] OR ""osteosarcoma""[All Fields] OR (""bone""[All Fields] AND ""sarcomas""[All Fields]) OR ""bone sarcomas""[All Fields]) AND (""disialoganglioside""[All Fields] OR ""sialogangliosides""[Supplementary Concept] OR ""sialogangliosides""[All Fields] OR ""disialogangliosides""[All Fields]) AND ""GD2""[All Fields]" |

| **Database 2**: Embase  **Coverage**: Database inception to 7 January 2026  **Search date**: 7 January 2026 | | |
| --- | --- | --- |
| **Search Number** | **Query** | **Search Details** |
| 1 | Osteosarcoma and GD2 | "('osteosarcoma'/exp OR osteosarcoma) AND gd2" |
| 2 | Bone sarcomas and GD2 | "bone AND sarcomas AND gd2" |
| 3 | Pediatric sarcomas and GD2 | "pediatric AND sarcomas AND gd2" |
| 4 | Ewing sarcoma and GD2 | "ewing AND sarcoma AND gd2" |
| 5 | Ewing sarcoma and anti GD2 | "ewing AND sarcoma AND 'anti gd2'" |
| 6 | Osteosarcoma and anti GD2 | "osteosarcoma AND 'anti gd2'" |
| 7 | Pediatric sarcomas and anti GD2 | "pediatric AND sarcomas AND 'anti gd2'" |
| 8 | Bone sarcomas and anti GD2 | "bone AND sarcomas AND 'anti gd2'" |
| 9 | Bone sarcoma and Disialoganglioside GD2 | "bone AND sarcoma AND 'disialoganglioside gd2'" |
| 10 | Pediatric sarcomas and Disialoganglioside GD2 | "pediatric AND sarcomas AND 'disialoganglioside gd2'" |
| 11 | Osteosarcoma and Disialonganglioside GD2 | "osteosarcoma AND 'disialoganglioside gd2'" |
| 12 | Ewing sarcoma and Disialoganglioside GD2 | "ewing AND sarcoma AND 'disialoganglioside gd2'" |

| **Database 3**: Web of Science  **Coverage**: Database inception to 7 January 2026  **Search date**: 7 January 2026 | |
| --- | --- |
| **Search Number** | **Query** |
| 1 | Bone sarcomas AND GD2 |
| 2 | Osteosarcomas AND GD2 |
| 3 | Pediatric sarcomas AND GD2 |
| 4 | Ewing sarcoma AND GD2 |
| 5 | Ewing sarcoma AND anti-GD2 |
| 6 | Osteosarcoma AND anti-GD2 |
| 7 | Bone sarcomas AND anti-GD2 |
| 8 | Pediatric sarcomas AND anti-GD2 |
| 9 | Pediatric sarcomas AND Disialoganglioside GD2 |
| 10 | Bone sarcomas AND Disialoganglioside GD2 |
| 11 | Osteosarcoma AND Disialoganglioside GD2 |
| 12 | Ewing sarcoma AND Disialoganglioside GD2 |
